# Clinical Markers of Need for Surgery in Orbital Complication of Acute Rhinosinusitis in Children: Overview and Systematic Review

**DOI:** 10.3390/jpm12091527

**Published:** 2022-09-18

**Authors:** Elena Cantone, Eva Piro, Eugenio De Corso, Claudio Di Nola, Stefano Settimi, Giusi Grimaldi, Gaetano Motta

**Affiliations:** 1 Department of Neuroscience, Reproductive and Odontostomatological Sciences-ENT Section, University of Naples “Federico II”, 80131 Naples, Italy; 2 Fondazione Policlinico Universitario A. Gemelli IRCCS, Head and Neck Surgery-Otorhinolaryngology, 00168 Rome, Italy; 3 Otorhinolaryngology, Head and Neck Surgery Unit, Department of Mental and Physical Health and Preventive Medicine, Università degli Studi della Campania Luigi Vanvitelli, 80131 Naples, Italy

**Keywords:** orbital cellulitis, periorbital cellulitis, subperiosteal abscess, acute rhinosinusitis, children, FESS, orbital surgery, ophthalmoplegia, proptosis

## Abstract

Background: Although they can occur at all ages, orbital (OC) and periorbital cellulitis (POC) prevail in the pediatric population. Acute rhinosinusitis (ARS) is the most frequent predisposing factor of OC. Recent literature has suggested a medical management approach for OC and POC, with surgery reserved only for more severe cases. However, there is still a lack of consensus on the clinical markers of a need for surgery. The aim of this systematic review was to identify clinical markers of a need for surgery in children with OC. Our systematic review, in accordance with the Preferred Reporting Items for Systematic Review and Meta-Analysis (PRISMA) process, yielded 1289 articles finally screened. This resulted in 31 full texts that were included in a qualitative analysis. The results of this review suggest that in children aged over 9 years, large subperiosteal orbital abscesses (SPOAs), impaired vision, ophthalmoplegia, proptosis, elevated C-reactive protein (CRP) and absolute neutrophil counts (ANC), hemodynamic compromise, no clinical improvement after 48/72 h of antibiotic therapy, and a Chandler III score or higher are clinical markers of the need for surgery. However, most of the studies are observational and retrospective, and further studies are needed to identify reliable and repeatable clinical markers of the need for surgery.

## 1. Introduction

Orbital complications of sinusitis in children are usually classified based on anatomic location. Cellulitis that is mainly anterior to the orbital septum, usually following an injury or due to the spread of a local infection, is called POC, whereas cellulitis posterior to the orbital septum, usually as a complication of rhinosinusitis, is called OC [1].

Although they can occur at all ages, POC and OC prevail in the pediatric population [1,2,3,4]. Given the difficulty in distinguishing these two entities, especially in children, POC and OC are often considered together in hospitalized children [1]. In clinical practice, otolaryngologists mainly deal with orbital forms. Among predisposing risk factors for OC, the most frequent include rhinosinusitis, ocular surgery, orbital trauma, and orbital foreign bodies [1]. For instance, ARS is responsible for 66–82% of cases of orbital infections and the acute ethmoiditis represents the most common sinus site linked to the OC in children. Due to the potential serious outcomes of OC, its management constitutes a medical and surgical emergency [2].

The most common symptoms and clinical signs indicative of OC are proptosis, ophthalmoplegia, and reduced extraocular mobility. However, these symptoms do not completely differentiate between OC and SPOA, which is a more severe complication, often requiring surgery. SPOA is a collection of purulence between the periorbita and the lamina papyracea, adjacent to the infected paranasal sinuses [3]. Generally, children are admitted to the general pediatric inpatient unit, with consultation from otolaryngologists, ophthalmologists, and other specialty services [1]. The consultation with an otolaryngologist is of utmost importance, in addition to a timely and justified execution of a computed tomography (CT) imaging study. Notwithstanding, the indications for CT to exclude OC from needing surgery are still not well established [1,3]. Recent literature suggests medical management for POC, OC, and for most patients with a SPOA, with surgery reserved for children with orbital abscess (OA) and cavernous sinus thrombosis [1].

Estimates of OC incidence range from 1.6 to 6 per 100,000 in pediatric populations, and orbital and intracranial complications of OC were documented in 0.7% of children presenting to emergency departments with the diagnosis of bacterial ARS [1,5]. Literature data have reported that 1.3–5.6% of ARS results in OC, with a higher incidence in the pediatric population under the age of 10, commonly attributed to an immature immune system [6]. Children are particularly prone to OC, not only for the immaturity of their immune system, but also because pediatric bones are thinner and more porous, and suture lines have not ossified in the manner of adult bones [7]. In addition, the thin lamina papyracea, naturally dehiscent, is the only bony border between the orbit and ethmoid sinuses and the communication with the orbit and cavernous sinus through a valve-free pathway might favor the occurrence of microbial accesses [7]. Rhinogenic orbital inflammation mainly affects children aged from 5 to 10 years and are the most frequent orbital complications of ARS. In addition, pansinusitis predominates in comparison with isolated inflammation of the paranasal sinuses (60–80%) [8]. From an epidemiological point of view, Cohen et al. described 76 children (67% males) with a diagnosis of OC and a mean of 31.9/100,000 admissions per year (range 0–72.3/100,000). The population ranged from 0.7 to 15.2 years, and the majority were younger than 9 years (63%), with a predominance (20%) of patients aged 1–2 years [9].

Etiological agents of OC in children are different from those in adults. Polymicrobial inflammation characterized by the presence of anaerobes is the most common type in adults, whereas *Streptococcus pneumonia* is one of the most common pathogens found in children with rhinosinusitis in the prepneumococcal vaccine era [8,10].

However, previous studies found no significant differences in the annual hospitalization rate before and after the introduction of the pneumococcal conjugate vaccine in 2010; this observation may suggest that *Streptococcus pneumonia,* as a causal pathogen of orbital cellulitis, was not high enough to affect any change [9]. *Haemophilus influenza*, and *Moraxella catarrhalis* are also described as etiological agents in the pediatric population [8]. According to Quintavilla-Dieck et al. the predominant organism group cultured in surgical children suffering from superior pediatric orbital SPOA was *Streptococcus anginosus (SA)* [11].

From a clinical point of view, OC children typically have periorbital erythema edema, rhinorrhea, headache, fever, ophthalmoplegia, impaired extraocular mobility, and proptosis [3]. OC and POC are diagnosed clinically when proptosis, ophthalmoplegia, or pain with eye movements are present; when the clinical examination is limited, complete blood counts (CBCs) and inflammatory markers (CRPs) are useful [1].

CT scans with contrast represent the standard for the diagnosis of OC and are useful to characterize not only the extent of infection in the periorbital region and the paranasal sinuses, but also the presence of an abscess [1]. However, although CT scanning is a valuable diagnostic tool, it does involve exposure to radiation. It is widely accepted that the radiation exposure from head and neck CT scans, especially in the pediatric population, can lead to an increased risk for malignancy, and thus should be selectively used, with strict indications [3]. The current CT classification of rhinogenic inflammatory complications involving the orbit is based on Chandler’s score, which is the most used classification system. There are five classes, including periorbital (preseptal) cellulitis (Chandler criteria I) and orbital cellulitis (Chandler criteria II), with the further three classes defined by the presence of a subperiosteal abscess (SPOA) (Chandler criteria III), orbital abscess (OC) (Chandler criteria IV), and cavernous sinus thrombosis (Chandler criteria V) [12]. According to the literature, CT is required in cases of intracranial complications, or when signs and symptoms of OC persist over a period of 24–48 h despite conservative therapy. However, a high proportion of patients with advanced Chandler’s classification, and mainly those with SPOA, might lack pathological ocular signs [3]. Repeat CT imaging is indicated only in cases of worsening vision or no clinical improvement within 24–48 h of initiation of therapy [13]. Generally, MRI has been useful in cases of intracranial complications of orbital cellulitis, including the cavernous sinus, and of negative CT scans, despite symptoms being concerning for abscess formation [14].

So far, there is still a lack of consensus on the clinical markers of a need for surgery. Data from recent studies are generally in favor of conservative treatment, but surgical drainage is usually recommended for OC, especially in the absence of improvement under medical treatment for 48 h, in more advanced Chandler stages, or in the suspicion of visual impairment [3].

The aim of this systematic review was to identify reliable clinical markers of the need for surgery in children with OC and POC.

## 2. Materials and Methods

### 2.1. Search Strategy

This systematic review was conducted in accordance with the Preferred Reporting Items for Systematic Review and Meta-Analysis (PRISMA) [15] process to identify published experimental and clinical articles about OC and POC and surgery published from 2002 to 2022. Papers prior to this period were included only if they were particularly relevant. Literature searches were performed in July 2022. Manuscripts were screened primarily by Ovid Medline and EMBASE and from other sources (PubMed Central, Cochrane review, Web of Science, and Google Scholar).

The authors focused on experimental and clinical studies matching the term as follows: ((orbital cellulitis) OR (periorbital cellulitis) OR (subperiosteal abscess) OR (preseptal cellulitis) OR (preseptal complication)) AND ((children) OR (childhood) OR (pediatric)) AND ((biomarker) OR (marker) OR (predictor)) AND ((surgery) OR (therapy)).

### 2.2. Study Selection

In the first screening, authors read the title and abstract of articles, being as inclusive as possible while making selections. The abstracts were screened independently by two authors. Any disagreements were resolved by consensus. Inclusion criteria, established before the selection of relevant studies, were primary research (including descriptive studies, observational studies, randomized trials, and basic science articles) addressing acute rhinosinusitis and OC, POC, or SPOA in children. Furthermore, we questioned whether clinical markers of a need for surgery exist. We excluded secondary research studies (e.g., review articles or systematic reviews), case studies, newspaper articles, lectures, letters, comments, personal narratives, consensus conferences, and editorials. Only articles with full text available were included. Additional studies were manually identified from the reference lists of retrieved literature. We excluded secondary research studies and all the articles that did not meet the inclusion criteria or deal directly with the issue investigated. We included only English language peer-reviewed papers.

## 3. Results and Discussion

**Figure 1:**
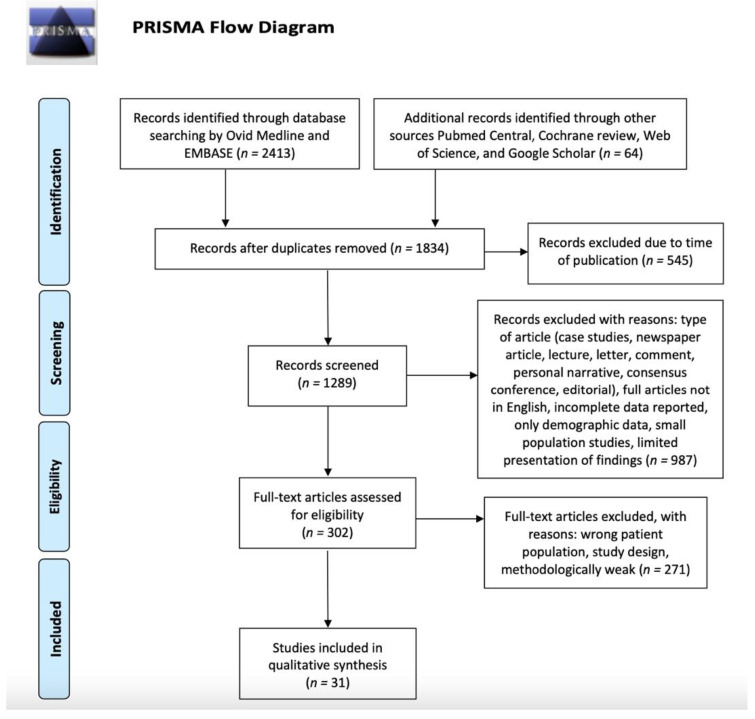
Flowchart of article search and selection according to the PRISMA criteria.

Details of the systematic search are shown in Figure 1. In total, our search yielded 1834 articles after duplicates were removed. We excluded 545 articles due to the time of publication and 1289 records were screened. A total of 1258 articles were excluded due to the type of article, wrong population, study design, or meeting other exclusion criteria. This resulted in 31 publications that were included. No studies were included in a quantitative synthesis (meta-analysis).

Medical and surgical care of patients with acute complicated rhinosinusitis is heterogeneous, with no clear indications for surgery in early-stage disease. Indeed, there is still a lack of consensus on the markers for surgery [7]. So far, no studies have been published to guide antibiotic therapy (oral or intravenous), antibiotic selection, or duration of therapy. Generally, empiric antibiotics are used to treat both Gram-positive and Gram-negative bacteria. Intranasal steroids (INS), intranasal decongestants, and intranasal saline are frequently used, although there are conflicting guidelines on whether these agents are useful [1].

Certainly, OC in children is not an absolute indication for immediate surgery [9]. Thus, antibiotic therapy is the mainstay of conservative management and in the early stages, empirical antibiotic therapy is generally initiated. Then, based on the culture and sensitivity results, broad-spectrum antibiotics are used [8]. In cases of negative swabs and blood cultures, a recent study supported the use of ceftriaxone in combination with metronidazole [4]. Generally, monotherapy (aminopenicillin or cephalosporin) is sufficient for the treatment of preseptal inflammation [8]. Medical management with intravenous antibiotics has been shown to be appropriate for children with complications limited to cellulitis or small SPOAs [6]. Furthermore, nasal mucosa toilets may facilitate the drainage of secretions through the nose [4]. The administration of intranasal decongestants and corticosteroids correlated with a smaller percentage of children, with or without periorbital abscesses, who required progression to surgery [4]. In addition, systemic or local corticosteroids can be used—albeit conflicting opinions exist [8]. A very recent study by Lahmini adapted antibiotic treatment and multidisciplinary care, which rendered surgery rarely necessary [16].

Therefore, in the light of the recent literature data, we can affirm that, although there are no clear indications on the type of molecule, on the type of administration, and on the duration of therapy, antibiotic therapy remains the first treatment choice, especially in the mild–moderate form of OC. Empirical therapy is generally initiated, then, after swab or culture, the most suitable molecule can be administrated. Other drugs, although commonly used, do not appear to be supported by sufficiently extensive studies to recommend their use.

Although, the endoscopic approach is preferred, the external approach can be considered in the case of recurrent or persistent abscesses, especially if they are in the upper or the lateral part of the orbit [3,6,8]. Decompression of one or more orbital walls may be necessary if orbital pressure remains elevated [17].

According to Cohen et al. characteristics associated with successful conservative management are normal vision, absence of ophthalmoplegia, being under 9 years of age, pansinusitis, and no proptosis [9]. However, when imaging reveals the presence of air bubbles in the subperiosteal space or in the intraconal space, early signs for the development of an abscess, as well as signs of systemic involvement, such as elevated CRP levels and hemodynamic compromise, may be early clinical markers of the need for surgery [9]. Again, Bulbul et al. [18] showed that a high CRP value could be used to predict orbital involvement; the authors suggested that SPOAs could be treated with medical treatment with close clinical and imaging follow-up. According to several authors, features that increase the likelihood of surgical management are being of an age above 9 years, the presence of proptosis, extraocular muscle restriction, elevated intraocular pressure, volume of SPA (>1.25–1.5 mL), impaired visual acuity, progression of local and systemic signs and symptoms (despite adequate conservative management for more than 24 h), and no local improvement after 48–72 h of antibiotic therapy [8,11]. In a recent study by Jiramongkolchai, patients aged 9 years and above were significantly more likely to have surgery compared to patients of less than 5 years of age. In addition, acute ethmoid sinusitis and acute pansinusitis were associated with the highest likelihood of surgery [13].

According to McDermott et al., if no improvement is noted in Chandler I or II pediatric patients after 24 to 48 h of intravenous antibiotics, including ampicillin sulbactam, surgery should be performed. Overall, in this study, when patients were started on a regimen containing ampicillin–sulbactam, a bactericidal agent with Gram-positive, Gram-negative, and anaerobic coverage, there was a decreased risk of need for surgery. This finding was consistent in the Chandler I to II children. Similar significance was not found in Chandler III patients [7]. Sciarretta et al. [2] suggested medical management as the main treatment for both preseptal and postseptal orbital cellulitis. Nevertheless, according to the authors, there was no universally accepted guideline for the treatment of SPOAs. Surgery should be considered in children not responding to medical management, or in cases of visual deterioration. Another study from Turhal pointed out that, despite most patients being responsive to medical therapy, radiologically proven abscesses required surgical intervention when there was loss in vision, limitation in ocular movement, or unresponsiveness to aggressive medical therapy [19]. In a recent study by Mabrouk, in Chandler I and II patients, the treatment was exclusively medical and in Chandler III, IV, and V, surgery could be indicated in association with medical treatment. In this study, fever, exophthalmos, positive CRP, age, and white blood cell count were not associated with more severe lesions in the CT scan. According to the authors, these parameters could not predict surgical indication [20].

In the study from Caldeira Santos et al. most patients with SPOAs responded well to conservative management and clinical factors for determining the need for surgery. These consisted of daily evaluations of neurologic symptoms, visual acuity, grade of proptosis, limitations and pain with eye movements, and pupillary reflexes, and neurologic exams [21].

According to Tzelnick et al. [22] children failing conservative treatment or presenting with abscesses in the acute phase required surgery. In addition, elective surgery or prophylactic antibiotic treatment should be strongly considered in patients with recurrent POC if an underlying etiology may not be uncovered.

Sansa-Perna limited the surgical approach by endonasal endoscopy to the abscesses located on the roof of the orbit or on the lateral wall, whereas the 4.2% of the total number of admitted children had surgery due to their orbital complications in the study by Dennison [23,24]. Ask et al. found that the lack of improvement in symptoms or the severity of symptoms in a cohort of 21 children required early CT scans, but only one child needed surgery for postseptal complications [25]. Wan et al. suggested that SPOAs, OC, and orbital abscesses should be treated by surgery, which should be performed in time to avoid causing more serious irreversible complications [26]. Rudloe, in addition to the known high-risk findings of ophthalmoplegia and proptosis, or in the absence of these signs, showed that an absolute neutrophil count of 10,000 cells/L also represented a high-risk category for surgery [27]. According to Deutschmann et al., infections by *SA* are significantly more likely to cause more severe intracranial complications and neurologic deficits and to require surgery [28]. In the study by Todman, in the case of multiple foci of bacterial infections, for children aged >9 years or who are immunodeficient, surgery should be considered if there is no improvement after >48 h following parenteral antibiotic treatment, as well as the presence of SPOAs and OC [29]. According to the authors, volumes <1250 mm^3^ did not require surgical management, whereas frontal sinusitis requiring surgical intervention always had concurrent SPOA volumes of ≥1250 mm [29]. In a recent paper, McCoy et al. [30] demonstrated that abscesses with a size of 0.510 cm^3^ or larger in any location were a relative indication for surgery. Thus, the volume of an SPOA seems to be one of the most important criteria in determining medical versus surgical management; many authors reported that SPOA volume is a significant prognostic factor for surgery, but the cut-off values above which surgery is required vary widely, from 0.48 mL to 3.8 mL. However, most authors strongly recommend the early surgical treatment of any SPOA of >500 mm^3^ (~0.5 mL) [31]. According to Rahbar et al., another marker for surgery is the proptosis; indeed, the probability of surgery is 92% for 2 mm of proptosis [32]. In the very recent study by Sorotzki, having three or more high-risk presenting symptoms was significantly associated with undergoing surgery [33]. For Presutti et al., immediate surgery was indicated not only in children with large SPOAs or OAs, but also in immunocompromised patients. Moreover, any worsening in the ophthalmological function must be carefully considered as a landmark in candidacy for surgery [17].

Persistent phlegmon and cellulitis despite intravenous antibiotics is an indication for surgery in the study by Tritt [34]. For Shifman, severe ocular presentation remained a classic indicator for surgery [35].

Markers for surgery in the study by Anosike were >5 years of age, proptosis, diplopia, or an SPOA ≥ 20 mm [36]. The presence of cellulitis/abscess of the face was the strongest predictor of multiple sinus procedures in the study by Villwock [6].

Jabarin pointed out that proptosis and ophthalmoplegia, higher neutrophil count, older age, and a malignant disease progression necessitating broad-spectrum antibiotic use are reliable predictors of an orbital abscess and surgery [3].

However, Dannison did not identify a single specific clinical marker that predicted the development of a severe complication [10].

Orbital involvement, such as pain in eye movements and proptosis were markers of the need for surgery in the study by Cürebal [37]. According to Mahalingam, there has been growing evidence to support the conservative management of such patients and surgery is increasingly reserved for those who deteriorate despite medical management with risk to vision, or those with neurosurgical complications [15]. While Martins et al. did not find markers for needing surgery, Gavriel suggested that children presenting with significant or progressing ocular findings or failure to improve after 48 h of medical therapy, together with an abscess volume of more than 0.5 mL, a length greater than 17 mm, and a width greater than 4.5 mm, should be strongly considered for surgical drainage [38,39].

Overall, the surgical therapy represents a second therapeutic step.

However, being of more than 9 years of age, the presence of large abscesses, and having impaired vision, ophthalmoplegia, proptosis, elevated CRP, no clinical improvement after 48/72 h of antibiotic therapy, and a Chandler III score or higher are early guides for the choice of surgery.

Surely, a watchful wait for of 48–72 h is always advisable in any case. Furthermore, careful screening of patients, not only by clinical and instrumental study but also by laboratory tests in order to choose candidates for surgery, is mandatory.

Although the therapeutic indications from the literature represent an essential guide for choosing the best treatments, we cannot overlook the fact that each patient is different from the others.

Therefore, from the point of view of precision medicine, therapeutic choices should always be customized with respect to the single patient, which is unique.

Table 1 provides a résumé for the articles included in the qualitative analysis and summarizes the most relevant results by pointing out the markers for surgery.

## 4. Conclusions

Based on the literature analyzed, we might affirm that the markers for surgery in children with OC are being over the age of 9 years and having large SPOAs, impaired vision, ophthalmoplegia, proptosis, elevated CRP and hemodynamic compromise, no clinical improvement after 48/72 h of antibiotic therapy, and a Chandler III score or higher. However, although most of the studies are observational (so are only able to identify association rather than causation) and retrospective (so are limited by the validity of diagnostic codes and the information documented in the medical records), this review provides interesting insights and draws useful conclusions, despite further studies being needed.

## Figures and Tables

**Table 1:** Articles included in the qualitative analysis and reviewed.

Authors (Years)	Type of Study	Patients (n)	Methods	Results	Clinical Markers of Need for Surgery
Choen, 2019 [9]	RCS	76	2 groups (<9/>9 years) and treatment strategy.	Conservative treatment of just antibiotics was administered to 76% of the cohort, with abscess drainage for the remainder.	Impaired vision, ophthalmoplegia, >9 years, no pansinusitis, proptosis, air bubbles in the subperiosteal space or in the intraconal space, elevated CRP, and hemodynamic compromise.
Bülbül, 2022 [18]	R	123	Findings of preseptal and orbital cellulitis groups were compared. The risk factors for the development of orbital involvement were analyzed.	Rhinosinusitis was the most common predisposing factor in the development of preseptal cellulitis and orbital cellulitis. Orbital involvement was present in 9.8% of the patients.	High CRP value could be used to predict orbital involvement.
Chrobok, 2019 [8]	R	292	Patients treated before functional endonasal surgery vs. patients treated by functional endonasal surgery as standard surgical method.	Most patients were treated conservatively; surgery was indicated in 1/3. Endoscopic endonasal approach.	SPOA and OA, impaired visual acuity, progression of local and systemic signs and symptoms despite adequate conservative management for more than 24 h, or no local improvement after 48/72 h of antibiotic therapy.
Quintanilla-Dieck, 2016 [11]	RCS	52	Imaging characteristics and treatment between superior and medial subperiosteal abscess	Superior subperiosteal abscess may present with more advanced disease, leading to a higher rate of characteristics such as proptosis, hypoglobus, and intraorbital air.	SPOA with proptosis, hypoglobus, and intraorbital air factors predispose to surgical drainage. Abscess volume is the most predictive of surgery (>0.67 mL).
Jiramongkolchai, 2020 [13]	R	10,148 children and adults	Trend of surgery over time and patient- and hospital-level factors associated with surgery from state inpatient databases	13% of children admitted for orbital cellulitis underwent surgery	>9 years, ophthalmologic comorbidities, and conjunctival edema
McDermot, 2020 [7]	Case series with charts review	168	Demographics, disease characteristics, in-hospital management, and outcomes were recorded and analyzed	49% surgery and 36% medical therapy followed by surgery. 83% of Chandler III received surgery	Chandler III and SPOA
Sciarretta, 2017 [2]	R	57	Data collection	Medical management was the main treatment for both preseptal and postseptal OC	SPOA
Thural, 2017 [19]	R	55	Data collection	Hospitalization in SPOA was higher than PSC. Conservative therapy was an effective method for PSC and most cases of OC	Nonresponsive to medical treatment within 48 h, ophthalmoplegia, and reduction in the visual acuity.
Mabrouk, 2020 [20]	CSS	39	Chandler I and II vs. Chandler III, IV, and V subgroups	Fever, exophthalmos, ophthalmoplegia, positive CRP, age, and white blood cells count were not associated with more severe lesions in the CT scan.	Orbital abscess
Caldeira Santos, 2019 [21]	R	122	Data collection	Fever, photophobia, ocular pain, painful eye movements, proptosis, rhinorrhea, and vison impairment were related with OC. Leukocytosis was present in 34.4% with OC.	Visual acuity, grade of proptosis, limitation and pain with eye movements, pupillary reflex, and neurologic exam.
Tzelnick, 2019 [22]	R	14	Data collection	Patients responded well to intravenous antibiotics, both during the primary and recurrent events. Surgery in patients failing conservative treatment or presenting with abscess formation.	Patients failing conservative treatment or presenting with abscess formation
Sansa-Perna, 2020 [23]	R	21 children and adults	Data collection	A CT scan was performed in all patients and the cases of subperiosteal or orbital abscess were treated surgically	Subperiosteal or orbital abscesses
Dennison 2019 [24]	R	215	Data collection	Postseptal OC occurred in 29 cases (13.5%) and surgery was necessary in 9 (4.2%).	Orbital complications
Ask, 2016 [25]	R	213	Data collection	CT scans were performed to deterioration of, or lack of improvement of status and/or symptoms and severity of the symptoms	CT verified postseptal complications and only one child needed surgery.
Wan, 2016 [26]	R	31	Data collection	16 patients were cured by conservative therapy and 15 patients by ESS.	No improvement after 48 h, orbital SPA, motility disorders of eyeball, or decreased vision
Rudloe, 2010 [27]	R	918	Data collection	Proptosis and/or pain or limitation of extraocular movements were at high risk for intraorbital abscesses, yet many did not have these predictors.	Orbital abscess, absolute neutrophil count—ANC of 10,000 cells/L, periorbital Edema, <3 years, previous antibiotic use, and Chandler III score or higher
Deutschman, 2013 [28]	RCS	50	medical records identified patients	Infection by the *S. Anginosus* were more severe and likely to require neurosurgical intervention with development of long-term neurologic deficits.	* S. Anginosus * infection
Todmann, 2011 [29]	R	29	Chart review	27.6% surgery and 72.4% medical therapy. The mean volume of abscesses needing surgery were larger.	Multiple foci of bacteria, >9 years, immunodeficiency, no improvement > 48 h after parenteral antibiotic treatment, SPA, and orbital cellulitis
McCoy, 2012 [30]	R	108	Data collection	With an abscess volume of 0.510 cm^3^, there was a sensitivity of 71.2% and a specificity of 84.4% for needing surgical drainage	Abscess size of 0.510 cm^3^ or larger
Rahbar, 2001 [32]	R	19	CT scans were reviewed	Probability of surgery was 92% for 2 mm of proptosis	Proptosis
Sorotzki, 2022 [33]	RCS	95	CT timing and results	Having three or more high-risk presenting symptoms was associated with a greater likelihood of surgical intervention	Number and severity of presenting complaints
Tritt, 2018 [34]	R	66	Number of days of intravenous antibiotics, complications, and need for admission.	2 children developed complications and one child underwent surgery.	Phlegmon and cellulitis despite intravenous antibiotics
Shifman, 2022 [35]	R	94	Clinical, laboratory and radiology characteristics, management, microbiological data, and outcomes were collected.	Children aged older than 9 years presented with markedly elevated inflammatory markers, i.e., leukocytosis and CRP.	Severe ocular presentation
Anosike, 2022 [36]	RCS	220	charts for demographic characteristics, clinical features, management, and outcomes	Although MRSA was rare, empiric vancomycin use was high. Treatment failure was uncommon in patients who received ≤ 2 weeks of therapy, suggesting that shorter durations are adequate in some patients.	>5 years of age proptosis, diplopia, or an SPOA ≥ 20 mm.
Villwock, 2020 [6]	KID	15,260	Frequency of sinus procedures in relation to pediatric age cohort were noted (<8 years vs. 9–20 years).	Sinus surgery in 7.2% of cases—with patients over 8 years having a 2.8-fold increase compared to younger patients.	Presence of cellulitis/abscess of the face was the strongest predictor of multiple sinus procedures
Jabarin, 2018 [3]	RCS	123	Age, gender, symptoms, physical findings, white blood, CRP levels, CT findings, treatment before and during admission, and surgical treatment	53 had SPOA	Proptosis and ophthalmoplegia, higher neutrophil count, older age, and a malignant disease progression necessitating broad-spectrum antibiotic use are reliable predictors of an orbital abscess.
Dannison, 2021 [10]	RCS	310	Age, gender, date of admittance, presence of redness and/or swelling around the eye, maximum CRP level, WBC, radiology results, surgery, and number of days with IV antibiotics.	1.3 surgeries per 100,000 per year. The percentage of admitted children that had surgery increased with age.	Not found
Cürebal, 2019 [37]	R	29	Age, gender, length of hospitalization, admission complaint, clinical features, complete blood count, CRP, radiology findings, blood culture results, antibiotic use and duration, and prognosis.	An accurate clinical approach and rapid treatment can prevent the spread of infection and avoid serious complications.	Orbital involvement, such as pain in eye movements, and proptosis.
Mahalingam, 2020 [4]	P	143 children and adults	Data collection	16.7% of children required surgery; ceftriaxone–metronidazole was associated with reduction in N of patients requiring surgery.	Chandler III and IV
Martins, 2021 [38]	R	55	Data collection	60% was treated medically and 40% required surgery. 12.73% developed complications. Higher ANC was found in patients with subperiosteal abscess.	Not found
Gavriel, 2011 [39]	R	95	Clinical and radiological parameters	Statistically significant larger abscesses in the surgically treated group were noted	Significant or progressing ocular findings, failure to improve after 48 h of medical therapy, together with an abscess volume of more than 0.5 mL, a length greater than 17 mm, and a width greater than 4.5 mm, should be strongly considered to have surgical drainage.

Abbreviations. R: retrospective study; RCS: retrospective cohort study; CSS: cross-sectional study; P: prospective study; KID: Kids’ Inpatient Database.

## Data Availability

Data are available upon reasonable request.
